# Power, Validity, Bias and Robustness of Family-based Association Analysis Methods in the Presence of Linkage for Late Onset Diseases

**DOI:** 10.1111/j.1469-1809.2008.00475.x

**Published:** 2008-11

**Authors:** J Nsengimana, J H Barrett

**Affiliations:** Section of Epidemiology and Biostatistics, Leeds Institute of Molecular Medicine, University of Leeds, Wellcome Trust Brenner Building, Level 5, St James's University, HospitalBeckett Street, LS9 7TF, Leeds, UK

**Keywords:** Extended sibships, conditional logistic regression, robust variance, simulation

## Abstract

This simulation-based report compares the performance of five methods of association analysis in the presence of linkage using extended sibships: the Family-Based Association Test (FBAT), Empirical Variance FBAT (EV-FBAT), Conditional Logistic Regression (CLR), Robust CLR (R-CLR) and Sibship Disequilibrium Test (SDT). The two tests accounting for residual familial correlation (EV-FBAT and R-CLR) and the model-free SDT showed correct test size in all simulated designs, while FBAT and CLR were only valid for small effect sizes. SDT had the lowest power, while CLR had the highest power, generally similar to FBAT and the robust variance analogues. The power of all model-dependent tests dropped when the model was misspecified, although often not substantially. Estimates of genetic effect with CLR and R-CLR were unbiased when the disease locus was analysed but biased when a nearby marker was analysed. This study demonstrates that the genetic effect does not need to be extreme to invalidate tests that ignore familial correlation and confirms that analogous methods using robust variance estimation provide a valid alternative at little cost to power. Overall R-CLR is the best-performing method among these alternatives for the analysis of extended sibship data.

## Introduction

Family-based association tests are tools of genetic association analysis robust to confounding effects of population stratification, a recurrent problem in classical case-control designs. The most popular family-based association test, the Transmission Disequilibrium Test or TDT, was originally designed to test for distortion in genetic transmission between parents and their affected offspring (Spielman et al. 1993). It has since been extended to other family and trait types by different authors (e. g. Allison, 1997; Spielman & Ewens, 1998; Clayton, 1999; Abecasis et al. 2000). Probably the most comprehensive extension is the Family-Based Association Test (FBAT) approach (Rabinowitz & Laird, 2000) implemented in the popular software of the same name (http://www.biostat.harvard.edu/~fbat). This method can be applied to both dichotomous and quantitative phenotypes in a variety of family types regardless of the structure of missing data, and multiple relatives are used while accounting for correlation in allele transmission among them (Lake et al. 2000). The ability to analyse families where parents are missing is of particular interest for late onset diseases where the parents of a proband have generally died. Accounting for correlation in allele transmission among siblings due to genetic linkage is important when multiple siblings are affected. In fact, while this correlation has no impact when the null hypothesis is ‘*no linkage and no association*’, it does induce spurious association when the null is ‘*linkage but no association*’ (Martin et al. 2000; Lake et al. 2000). Previously some authors had proposed to resolve the problem of residual familial correlation by selecting only one affected per family (Curtis, 1997), but this approach is clearly inefficient. Lake et al. (2000) proposed to account for residual familial correlation by using an empirical variance-covariance estimator in place of the classical variance-covariance matrix used in standard FBAT, giving a new test that they termed EV-FBAT (option –e in FBAT program).

Conditional logistic regression (CLR, available in most statistical software e.g. SAS, SAS Institute Inc., Cary, NC, USA, and STATA, StataCorp, 2005) is a traditional method of analysis of matched case-control studies in epidemiology. This is also appropriate for testing genetic association using familial data (Siegmund et al., 2000), since the within-family analysis provides protection against population stratification. In a simulation study, Siegmund et al. (2000) showed that, although standard CLR (using the Wald test) applied to families of multiple affected siblings is valid in the case of low genetic effect, it has an inflated type 1 error when the locus has a strong effect. They demonstrated that using the robust variance-covariance estimator (Rogers, 1993) in place of classical variance-covariance removes this effect of residual familial correlation. The new test derived by Siegmund et al. (2000) by applying robust variance-covariance will be referred to here as R-CLR (robust conditional logistic regression).

FBAT and EV-FBAT are dedicated to genetic association analysis while CLR and R-CLR are more general methods available in general software. They have important distinctive features such as the straightforward extension to haplotypes in FBAT which is unavailable for general programs (haplotypes have to be externally reconstructed to be analysed with CLR/R-CLR, see e. g. Nsengimana et al. 2007). On the other hand, additional risk factors and their interactions can be easily incorporated in the CLR/R-CLR approach, while they can only be indirectly considered in FBAT/EV-FBAT (by separate regression analysis and application of FBAT/EV-FBAT to residuals of this regression, see e. g. Lu & Cantor, 2007). In addition, CLR/R-CLR allow estimation of the size of genetic effect in terms of the odds ratio, which is not feasible in FBAT.

To our knowledge there is no published study comparing FBAT and EV-FBAT in a large range of family designs and disease models and showing the effectiveness and cost of empirical variance in removing the effect of residual familial correlation. Likewise, there is no published study comparing the power of FBAT/EV-FBAT and CLR/R-CLR approaches. Here we report on a simulation study comparing type 1 error rate, power, bias and robustness to model misspecification associated with these methods when testing for association in the presence of linkage using extended sibships. We consider a mixture of sibship sizes and structures (numbers of affected and unaffected siblings) in the absence of parental data. Because the true genetic model is generally unknown, it may be preferable to use a method that does not make any assumptions about the mode of inheritance. For this reason, we further compare the power of all these model-based tests (FBAT, EV-FBAT, CLR and R-CLR) under correct and incorrect models with the Sibship Disequilibrium Test (SDT, Horvath & Laird, 1998), a model-free sign test contrasting the number of sibships where a particular allele is more common in affected versus unaffected siblings. SDT has been shown to be less powerful than CLR and R-CLR under a correct genetic model (Siegmund et al. 2000) but we investigate whether SDT is still inferior when the model is misspecified.

## Methods

Five methods are compared: FBAT, EV-FBAT, CLR, R-CLR and SDT. Firstly the type 1 error rate of all five tests is evaluated using a variety of family designs and disease models and the power is compared wherever the size of the test is correct. Secondly, for tests requiring a genetic model specification (FBAT, EV-FBAT, CLR and R-CLR) robustness to model misspecification is assessed by calculating power assuming a dominant and a recessive model with data simulated using an additive model. Also, since there is generally no guarantee that the polymorphism under investigation is the actual disease locus, situations are considered in power estimation where they coincide and are maximally associated (D’ = 1.0) and where they are at a certain (small) distance from each other and linkage disequilibrium (LD) between them is weaker (D’ = 0.5). As CLR and R-CLR allow estimation of the strength of genetic effect, the bias of this estimate is evaluated when the tested locus is the disease locus and when it is a nearby marker. Nicodemus et al. (2007) have recently compared TDT-derived methods in an attempt to draw some guidelines on how to choose the appropriate test according to the data or how to design the study in order to apply a particular test. Unlike these authors who compared the methods that use different types of data, here the experimental design is fixed *a priori* as discordant sibships with missing parents, with the common situation of late onset diseases in mind. A mixture of sibship sizes are considered with variable numbers of affected and unaffected siblings. The family structures simulated are based on those found in a cardiovascular disease candidate-gene study (Nsengimana et al. 2007).

### Simulated Designs

A dichotomous disease outcome is considered, and for each design (Table 1) 10,000 replicates are simulated. For type 1 error evaluation, the marker and disease locus were linked but not associated, i.e. they were in linkage equilibrium. For most designs the recombination fraction θ was set to the most extreme value of zero, since the tighter the linkage the more inflation of type 1 error is expected. For the more extreme designs (12 and 13), where some inflation of type 1 error was seen (see Results), θ was varied between 0 and 0.5 to examine the effect of weaker linkage. For power estimation, two situations were considered: marker = disease locus and distance from marker to disease locus equals 50 kb (recombination rate θ= 0.0005, assuming 1 Mb ≈ 1 cM) with D’ = 0.5 (r^2^= 0.25). This level of LD at this distance was chosen because an average D’ of 0.50 has been observed at ∼50 kb in 19 randomly selected regions across the human genome (Reich et al. 2001). We fixed the distance between the marker and the gene locus because we defined the LD level in the parental generation, whereas the analysis is done in the offspring generation. The LD decreases between the two generations but the low distance chosen means that the decrease is negligible. In all designs, the marker and disease locus were biallelic and had equal minor allele frequency ranging from 0.10 to 0.50. At the disease locus, the susceptibility allele was the one with lowest frequency. The additive genetic model (on the logistic scale) was simulated, and genotype penetrances were varied from 0.10 to 0.90, giving overall population prevalence of the disease between 17 and 50% with population-attributable fraction (PAF) of the locus ranging between 5 and 80% and genetic odds ratio (GOR) of 1.3 to 9 per copy of variant allele (Table 1). These parameters were chosen to be consistent with a common disease model with small to high GOR from the locus of interest, the highest values being set to assess the behaviour of the tests in extreme situations.

**Table 1:** Designs simulated to assess type 1 error and power

Design	f_D_ (1)	P_DD_ (2)	P_ND_ (3)	P_NN_ (4)	GOR (5)	Prev (6)	PAF (7)
1	0.10	0.31	0.25	0.20	1.33	21	5
2	0.30	0.31	0.25	0.20	1.33	23	13
3	0.10	0.51	0.40	0.30	1.55	32	6
4	0.30	0.51	0.40	0.30	1.55	36	17
5	0.10	0.42	0.30	0.20	1.71	22	9
6	0.30	0.42	0.30	0.20	1.71	26	24
7	0.10	0.39	0.25	0.15	1.89	17	12
8	0.30	0.39	0.25	0.15	1.89	21	30
9	0.10	0.64	0.40	0.20	2.67	24	17
10	0.30	0.64	0.40	0.20	2.67	32	38
11	0.10	0.84	0.60	0.30	3.5	36	17
12	0.30	0.84	0.60	0.30	3.5	47	37
13	0.50	0.90	0.50	0.10	9	50	80

(1) Frequency of disease allele D and minor allele frequency at the marker locus; (2) Penetrance in homozygous carriers; (3) Penetrance in heterozygotes; (4) Incidence in non-carriers; (5) Genetic odds ratio per copy of disease allele; (6) Disease prevalence in the population (%); (7) Population-attributable fraction (%).

For power calculations, a total of 1,000 sibships were simulated with fixed proportions of various numbers of affected and unaffected siblings (Table 2) close to the proportions in our cardiovascular study. For type 1 error evaluation, larger sibships were considered (Table 3) to allow for a higher impact of familial correlation. The simulated data were analysed within the FBAT program for tests FBAT, EV-FBAT and SDT, while STATA v.9 (StataCorp, 2005) was used for CLR and R-CLR (testing for association using the Wald test). The simulation program (written in C) is available upon request.

**Table 2:** Number and structure of simulated sibships for power comparison

Sibship size	2	3	3	4	4	5	5
Affected	1	1	2	2	3	2	3
Unaffected	1	2	1	2	1	3	2
Number of sibships	200	450	200	50	60	20	20

**Table 3:** Number and structure of sibships simulated for type 1 error evaluation

Sibship size	5	5	6	6	6
Affected	3	4	3	4	5
Unaffected	2	1	3	2	1
Number of sibships	150	250	150	400	50

## Results

### Type 1 Error Rate

Because most genetic association studies involve testing multiple hypotheses, we report test size and power at the 0.001 level. In all the designs with GOR<2, all five tests have correct size as shown in Table 4. In all the designs with GOR>2, FBAT and CLR showed significantly inflated type 1 error, while SDT, EV-FBAT and R-CLR remained valid. For the two most extreme designs (designs 12 and 13), simulations were carried out with different distances between the marker and the susceptibility locus. In both designs, type 1 error inflation in FBAT and CLR was higher with tighter linkage (Table 4), remaining significant at a recombination fraction of 0.10 in design 12 (GOR = 3.5/PAF = 37%) and 0.20 in design 13 (GOR = 9/PAF = 80%).

**Table 4:** Type 1 error at level 0.001

Design (1)	PAF (%)	GOR	FBAT	EV-FBAT	CLR	R-CLR	SDT
1	5	1.33	0.0004	0.0005	0.0003	0.0006	0.0006
2	13	1.33	0.0005	0.0006	0.0008	0.0008	0.0005
3	6	1.55	0.0012	0.0008	0.0011	0.0010	0.0011
4	17	1.55	0.0012	0.0010	0.0007	0.0009	0.0006
5	9	1.71	0.0013	0.0009	0.0015	0.0011	0.0007
6	24	1.71	0.0010	0.0005	0.0012	0.0009	0.0004
7	12	1.89	0.0013	0.0007	0.0011	0.0007	0.0008
8	30	1.89	0.0015	0.0007	0.0014	0.0013	0.0008
9	17	2.67	0.0027**	0.0013	0.0022**	0.0015	0.0008
10	38	2.67	0.0027**	0.0014	0.0019*	0.0014	0.0009
11	17	3.5	0.0020*	0.0010	0.0025**	0.0013	0.0007
12							
θ= 0.0			0.0033**	0.0014	0.0026**	0.0015	0.0015
θ= 0.1			0.0021*	0.0010	0.0018*	0.0008	0.0006
θ= 0.2	37	3.5	0.0012	0.0005	0.0012	0.0007	0.0007
θ= 0.3			0.0009	0.0007	0.0014	0.0013	0.0010
θ= 0.4			0.0009	0.0008	0.0010	0.0011	0.0009
θ= 0.5			0.0011	0.0009	0.0009	0.0010	0.0004
13							
θ= 0.0			0.0046**	0.0011	0.0052**	0.0006	0.0008
θ= 0.1			0.0032**	0.0011	0.0029**	0.0013	0.0011
θ= 0.2	80	9	0.0017*	0.0010	0.0019*	0.0008	0.0013
θ= 0.3			0.0015	0.0010	0.0013	0.0009	0.0009
θ= 0.4			0.0013	0.0014	0.0014	0.0013	0.0010
θ= 0.5			0.0011	0.0010	0.0011	0.0012	0.0015

(1) Error 1 rate at increasing distance marker-disease locus for the two most extreme designs. In all other designs there is zero recombination rate between the marker and disease locus.

(*) Significant inflation at level 0.001<p<0.05

(**) at level p<0.001.

### Power Comparison when the Correct Model is Used

Power is compared between the 5 methods in the designs where they all showed correct test size (those with GOR<2, see Table 4). In order to assess any loss of power due to correcting for familial correlation for a stronger locus effect, the power of all tests is also compared in designs 9 and 10 where the tests using the standard variance-covariance matrix showed inflated type 1 error (designs with GOR = 2.67). As shown in Table 5, FBAT, EV-FBAT, CLR and R-CLR have comparable powers in all designs, while SDT is consistently less powerful. At the 0.001 significance level all tests have a poor power (<20%) in the design of weakest genetic effect (GOR = 1.33/PAF = 5%). Power rises however to over 56% (40% for SDT) in the same design with a more common disease allele (GOR = 1.33/PAF = 13%) when the marker locus coincides with the disease locus. At a GOR of 1.55 and allele frequency 30% (design 4), the power jumps to ∼97% (91% for SDT) when the marker is the disease locus (Table 5).

**Table 5:** Power at level 0.001 under the correct model (%)

Design (1)	PAF (%)	GOR	D'	FBAT	EV-FBAT	CLR	R-CLR	SDT (2)
1	5	1.33	1.0	16.6	15.9	16.9	16.4	9.9
			0.5	1.7	1.6	1.7	1.6	0.9
2	13	1.33	1.0	56.4	55.6	56.5	56.0	39.6
			0.5	6.2	6.0	6.3	6.1	3.9
3	6	1.55	1.0	60.5	59.3	61.2	60.1	42.5
			0.5	7.0	6.7	7.2	6.8	4.2
4	17	1.55	1.0	97.4	97.3	97.7	97.6	91.3
			0.5	25.4	24.6	26.3	25.6	15.8
5	9	1.71	1.0	88.5	87.9	89.0	88.4	76.8
			0.5	16.2	15.4	16.4	15.6	10.1
6	24	1.71	1.0	99.9	99.9	99.9	99.9	99.4
			0.5	50.6	49.7	51.4	50.0	35.9
7	12	1.89	1.0	98.2	98.1	98.2	98.2	94.5
			0.5	33.4	31.9	33.3	31.9	22.8
8	30	1.89	1.0	100	100	100	100	100
			0.5	74.6	73.3	74.8	73.6	59.7
9	17	2.67	1.0	100	100	100	100	100
			0.5	84.8	83.1	85.5	83.9	70.3
10	38	2.67	1.0	100	100	100	100	100
			0.5	99.2	99.2	99.3	99.2	97.1

(1) FBAT and CLR are not valid for the two last designs with GOR = 2.67 (see Table 4).

(2) SDT does not require model specification.

The power is lower when disease and marker loci do not coincide and the LD between them is 50% of the maximum. It remains lower than 35% for all designs with GOR<2 and variant frequency = 10% (designs 1, 3, 5 and 7), but it hits 99% (97% for SDT) in the strongest model analysed. In designs where GOR = 2.67, valid tests EV-FBAT and R-RCLR appear to remain as powerful as FBAT and CLR, indicating that there is little cost to power from using robust variance even for strong locus effects. However some drop of power would be expected when robust variance is used, and this is more apparent when more stringent significance levels are applied, moving the power away from 100%. As can be seen in Table 6, the power of standard and robust/empirical variance methods remain of the same magnitude in designs 4 to 8 but robust/empirical variance is associated with substantially lower power in designs 9 and 10. It also appears that at high GORs and low α values the R-CLR method is more powerful than EV-FBAT.

**Table 6:** Power at more stringent significance levels under the correct model (%)

Design	PAF (%)	GOR	α	D'	FBAT	EV-FBAT	CLR	R-CLR	SDT (1)
4	17	1.55	10^−5^	1.0	80.6	79.3	81.8	81.0	60.1
				0.5	3.7	3.3	4.0	3.7	1.9
6	24	1.71	10^−7^	1.0	88.0	86.6	89.1	87.5	69.9
				0.5	2.3	1.9	2.5	2.1	1.0
7	12	1.89	10^−5^	1.0	84.1	82.4	84.3	82.9	69.0
				0.5	5.7	5.0	5.7	5.0	2.8
8	30	1.89	10^−10^	1.0	90.0	87.4	90.8	88.1	69.5
				0.5	0.6	0.4	0.7	0.4	0.1
9	17	2.67	10^−12^	1.0	85.2	76.3	87.9	80.4	59.8
				0.5	0.2	0.1	0.2	0.1	0.1
10	38	2.67	10^−25^	1.0	80.9	62.0	88.4	71.0	44.4
				0.5	0.0	0.0	0.0	0.0	0.0

(1) SDT does not assume a genetic model.

### Estimating the Strength of Genetic Effect

CLR and R-CLR offer the possibility of estimating the genetic effect as a GOR. Along with true simulated and estimated GORs, Table 7 shows the proportion of simulations where the 95% confidence interval (CI) contains the true value. Both methods give the same estimate of GOR but have different CIs, as the only difference between them is the variance they use (i.e. standard *vs*. robust variance). In all the designs considered, CLR and R-CLR show an unbiased estimate of GOR and good CI coverage as long as the analysed marker is the disease locus (Table 7). However, as expected, when LD is reduced the estimate is biased and the 95% CI coverage is clearly poor. The bias and drop in CI coverage increase with the strength of true genetic effect and the variant allele frequency. The true value is contained in the 95%CI in only 3 simulations in 10,000 when GOR = 2.67, variant frequency = 30% and D’ = 0.5 (design 10).

**Table 7:** Estimated genetic odds ratio and confidence interval coverage

Design	PAF (%)	True GOR	D'	Estimated GOR	95CI for CLR (1)	95CI for R-CLR (1)
1	5	1.33	1.0	1.34	95.1	95.0
			0.5	1.17	80.6	80.5
2	13	1.33	1.0	1.34	94.7	94.7
			0.5	1.16	62.3	62.4
3	6	1.55	1.0	1.56	95.0	94.9
			0.5	1.27	59.4	59.5
4	17	1.55	1.0	1.55	94.9	94.8
			0.5	1.25	24.5	24.7
5	9	1.71	1.0	1.72	95.3	95.3
			0.5	1.34	48.4	48.7
6	24	1.71	1.0	1.71	95.0	94.9
			0.5	1.32	12.4	12.7
7	12	1.89	1.0	1.90	94.9	95.0
			0.5	1.43	35.7	36.3
8	30	1.89	1.0	1.90	94.9	94.9
			0.5	1.39	4.8	4.9
9	17	2.67	1.0	2.67	95.0	95.0
			0.5	1.71	5.5	5.6
10	38	2.67	1.0	2.68	95.3	95.3
			0.5	1.64	0.03	0.03

(1) Proportion (%) of simulations where the 95% confidence interval contains the true GOR.

### Power Comparison Under an Incorrect Genetic Model

The data simulated using an additive model were also analysed with four tests (FBAT, EV-FBAT, CLR, R-CLR) assuming dominant and recessive models. Under the dominant model, all four methods remain equally powerful (Table 8). Power is lower than when the correct model was used but remains higher or comparable to that of SDT (Tables 5 and 8). The largest drop in power is observed in designs with a common disease variant. For example, when the marker is the disease locus the power of the four tests drops from ∼56% to 37% in design 2, and where the marker is in LD with the disease locus (D’ = 0.5), the power drops from ∼74% to 54% in design 8. When the analysis model is recessive, the power decreases more sharply for all four tests and it falls below the power of SDT in all the designs. In contrast to dominant models, the most dramatic fall is observed in the designs with smallest disease allele frequency (Table 8) as one might expect. In design 7 (GOR = 1.89), the highest power in the four tests hardly reaches 14% where the power of SDT hits 94%.

**Table 8:** Power at level 0.001 using an incorrect model

Model (1)	Design	PAF (%)	GOR	D'	FBAT	EV-FBAT	CLR	R-CLR
Dominant	1	5	1.33	1.0	13.7	13.6	13.8	13.3
				0.5	1.6	1.5	1.6	1.5
	2	13	1.33	1.0	36.6	36.8	36.7	36.2
				0.5	3.7	3.9	3.7	3.6
	3	6	1.55	1.0	54.8	53.8	55.3	54.3
				0.5	6.1	5.8	6.2	5.8
	4	17	1.55	1.0	89.5	89.5	89.9	89.5
				0.5	15.4	15.0	16.0	15.6
	5	9	1.71	1.0	84.0	84.0	84.2	83.7
				0.5	14.0	13.4	14.2	13.4
	6	24	1.71	1.0	98.9	98.9	99.0	98.9
				0.5	33.0	32.7	33.2	32.2
	7	12	1.89	1.0	96.6	96.5	96.7	96.6
				0.5	29.0	28.1	29.2	27.9
	8	30	1.89	1.0	99.9	99.9	99.9	99.9
				0.5	54.2	53.9	54.3	53.0
Recessive	1	5	1.33	1.0	0.8	0.3	0.9	0.2
				0.5	0.2	0.1	0.4	0.0
	2	13	1.33	1.0	13.0	11.4	12.7	12.2
				0.5	1.4	1.1	1.4	1.2
	3	6	1.55	1.0	2.2	0.8	2.2	0.8
				0.5	0.5	0.1	0.5	0.1
	4	17	1.55	1.0	47.2	43.8	49.4	47.7
				0.5	4.5	3.9	4.8	4.3
	5	9	1.71	1.0	5.8	2.6	6.1	3.4
				0.5	0.7	0.3	0.8	0.4
	6	24	1.71	1.0	80.1	77.6	81.0	80.1
				0.5	10.9	9.3	11.0	10.5
	7	12	1.89	1.0	13.3	7.6	13.8	9.7
				0.5	1.5	0.6	1.4	0.7
	8	30	1.89	1.0	96.6	95.9	96.5	96.3
				0.5	21.2	18.2	20.9	19.7

(1) This is the analysis model. The simulated model is additive in all the designs.

## Discussion

Family-based association methods were originally designed to test for the presence of linkage or association. While testing this composite null hypothesis can be interesting in some instances, many candidate gene fine-mapping studies aim to search for association in a region where linkage has already been found or where it can be reasonably assumed. Therefore, the methods that can test association in families while accounting for linkage are valuable tools. In this study, we compare the performances of five methods of family-based association analysis: FBAT, EV-FBAT, CLR, R-CLR and SDT. Our study shows that in the case of low genetic risk all five tests have a correct type 1 error rate (Table 4). However, for genes with a GOR higher than 2, FBAT and CLR have an inflated type 1 error while their counterparts using robust variance estimation remain valid. For CLR and R-CLR, our observations confirm those made by Siegmund et al. (2000), although the two studies bear some differences. In their study, Siegmund et al. (2000) used 200 and 500 sibships of size 3 and 4, assessed test validity at GOR = 2 and 20 (log additive model) and used a nominal p-value of 0.05. They concluded that ‘*the genetic effect needs to be quite extreme before residual familial correlation due to linkage led to false positive inference*’ using CLR. In our study, we found that CLR is not valid (level 0.001) for a GOR of 2.67 which is far less extreme than 20. Our study demonstrates that familial correlation is not only influenced by the strength of the genetic effect, but also, maybe more critically, by the number of affected siblings within a family. Robust variance should therefore be recommended in the analysis of large sibships even when a modest genetic effect is suspected.

Our study of power confirms the results of Siegmund et al. (2000) and Hancock et al. (2007) that robust variance resolves the problem of familial correlation at little or no cost to power in CLR for most practical situations, and we make a similar observation with FBAT. However, when GOR is high, robust variance induced a substantial power loss to both FBAT and CLR at stringent significance levels (moving power away from 100%). Also, while EV-FBAT and R-CLR showed comparable power at level 0.001 in all simulated designs, R-CLR appears as the most powerful test at very low alpha values. Combined with the flexibility of this method (for example the ease of adding covariates, testing for interactions and estimating the strength of genetic effects), this result suggests that R-CLR is a better choice than EV-FBAT for the analysis of extended sibship data.

All the tests compared under incorrect genetic model specification have shown similar results: with the additive model simulated, a small drop in power when the dominant model is assumed and a sharper power decrease with the recessive model (Table 8). Unsurprisingly, the rarer the disease allele, the more pronounced the loss of power under the recessive model. In effect, only the homozygous affecteds contribute to the test, and their number is very small even for relatively common alleles. Although SDT was the least powerful method when the model is correctly specified in the other tests (Table 5), it is actually more powerful than any other test in all simulated designs when the analysis assumes (incorrectly) a recessive model. SDT may therefore be preferable to other tests in some instances or, alternatively, it can be used in addition to EV-FBAT or R-CLR. In such a case, a highly significant association found with SDT undetected by other approaches would suggest wrong model specification.

CLR and R-CLR provide precise estimates of the GOR when the marker is the disease locus. However, as expected, when the marker and the disease loci are distinct and are not maximally associated, the estimate of GOR is always biased and the 95% CI coverage can be very poor. The stronger the true genetic effect and the commoner the disease allele frequency, the higher the bias. Cordell (2004) reported a similar observation using a CLR method based on conditioning on parental genotypes (using parents and their affected offspring). The bias increased with the number of affected siblings and with the size of true genetic effect. More recently, Hancock et al. (2007) reported the same bias using R-CLR and generalised estimating equations. They showed that generalised estimating equations are even more biased than R-CLR, particularly for gene x gene interaction effects. In our study, the bias was influenced by LD, the true strength of genetic effect and the allele frequency. Missing parental genotypes do not appear to be a source of bias, since bias was also found when parents were used (Cordell, 2004; Hancock et al. 2007).

Our study has some limitations. First, this is not a comprehensive evaluation of all existing family-based association tests. There are now many methods, most of which were optimised for particular data configurations and are not necessarily perfectly comparable. We chose to focus on one general statistical approach (CLR) and one specialised genetic method (FBAT) both of which are very widely used for sibship data. Second, a comprehensive comparison would necessitate a larger spectrum of family structures, sample sizes and disease models (genetic effect, allele frequency, linkage disequilibrium, etc.), not all of which would be feasible to consider in one study. We chose instead to concentrate on one large mixture of family structures as observed in an actual study of affected and unaffected siblings with cardiovascular disease (Nsengimana et al. 2007). Although this design only represents one type of study, a mixture of family structures (sibship sizes + numbers of affected/unaffected) is common and is therefore in this context more realistic than the range of homogeneous designs often considered (e. g. Siegmund et al. 2000, Martin et al. 2000).

In conclusion, our simulations show that the genetic effect does not need to be extreme to invalidate tests that use standard variance estimators (CLR and FBAT). For more complex pedigrees EV-FBAT has clear advantages, but, based on power, validity and robustness to model misspecification, R-CLR provides a powerful suitable alternative for the analysis of extended sibships.
